# Supplementary material for: Characterization of the Gut Microbiome in Healthy Dogs and Dogs with Diabetes Mellitus
Source: Animals (Basel). 2023 Aug 1;13(15):2479. doi: 10.3390/ani13152479 (PMC10417117; doi:10.3390/ani13152479)
Supplement: Supplementary file 1 [file animals-13-02479-s001.zip › animals-2497678-supplementary.pdf]

Supplementary Materials

Figure S1. Alpha diversity between healthy and diabetic groups.

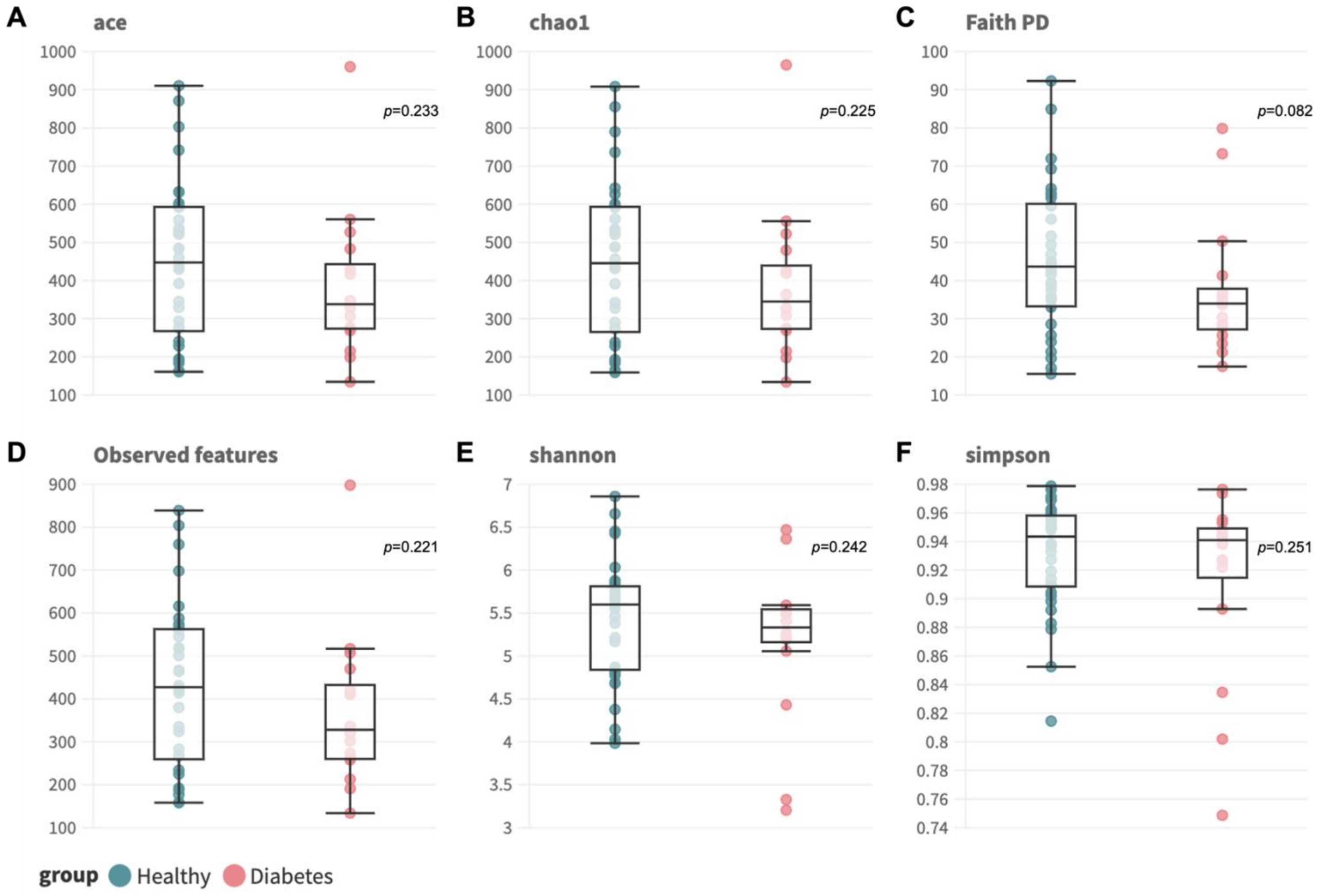

Table S1. Adonis test statistics of beta diversity.

Variable	Bray-Curtis	cosine	Hamming	Jaccard	gUniFrac	uwUniFrac	wnUniFrac	wUniFrac
**Age Group**	0.037	0.024	0.070	0.045	0.443	0.081	0.456	0.442
**Breed Size**	0.131	0.209	0.139	0.221	0.419	0.224	0.423	0.456
**Gender**	0.789	0.505	0.733	0.881	0.724	0.279	0.725	0.706
**Group**	0.027	0.114	0.271	0.002	0.251	0.001	0.243	0.233

*Abbreviations: gUniFrac = Generalized Unique Fraction Distances; uwUniFrac = Unweighted Unique Fraction Distances; wnUniFrac = Weighted Normalized Unique Fraction Distances; wUniFrac = Weighted Unique Fraction Distances

Table S2. Top 10 hub nodes across networks.

ASV	Taxon	Group	Degree
bb3055f753c1dd21f0137f2dd59d49ec	f__Helicobacteraceae; g__Helicobacter	Healthy	25
adc085fb68c6ca79a17867f7d5dfe238	f__Enterobacteriaceae; g__Escherichia-Shigella; s__Escherichia_sp.	Healthy	25
99acc08c0d8bfce4ad95740ddf504a83	f__Erysipelatoclostridiaceae; g__Erysipelatoclostridium; s__[Clostridium]_spiroforme	Healthy	24
3c72324085d7022a73a0bf57beb0b3fc	f__Lachnospiraceae; g__Lachnospiraceae_NK4A136_group; s__uncultured_bacterium	Healthy	24
7a35b8233e75ddc42a93f46704d53d1f	f__Enterobacteriaceae; g__Escherichia-Shigella; s__Escherichia_sp.	Healthy	23
a25ccb444dbbbc340eb5926283bf134b	f__Prevotellaceae; g__Prevotellaceae_Ga6A1_group; s__uncultured_bacterium	Healthy	23
46f019631ec39bbb20d30b4a3ae24704	f__Streptococcaceae; g__Streptococcus; s__Streptococcus_lutetiensis	Healthy	23
8c59053f20bd4788d68a45616fc58ee8	f__Lachnospiraceae	Healthy	22
a3a5f80c745d186df4adaeb7f1ef8a32	f__Bacteroidaceae; g__Bacteroides; s__Bacteroides_vulgatus	Healthy	22
5c8e65a01a47c84e85f83cac65a5e0b5	f__Peptostreptococcaceae; g__Peptostreptococcus; s__Peptostreptococcus_anaerobius	Healthy	22
3e48499d30bb1bc080c438249c940174	f__Pectobacteriaceae; g__Sodalis; s__bacterium_secondary	Diabetes	147
8f6b2d5f877dd8c9a342fab371c4d92f	f__Prevotellaceae; g__Alloprevotella	Diabetes	133
8c86daad090df502f91909b7794dd72a	f__Helicobacteraceae; g__Helicobacter	Diabetes	122
01ebe1bdf28b4b98908f3eb63287170f	f__Prevotellaceae; g__Alloprevotella	Diabetes	116
50f31ae556c607afe407e6610551f887	f__Lachnospiraceae	Diabetes	40
38ab081ee38e634b89bb6eaaba005e48	f__Lachnospiraceae; g__Blautia	Diabetes	38
851d8b0b44c78c7ce24c78b9f14b1d85	f__Bacteroidaceae; g__Bacteroides; s__Bacteroides_stercoris	Diabetes	34
d862ff0f8f5c9f39ae13f080aa680bb3	f__Lachnospiraceae; g__Roseburia; s__Roseburia_intestinalis	Diabetes	30
f7a1c2a7dc74eb24446faaa79f519ee6	f__Lachnospiraceae; g__Agathobacter; s__[Eubacterium]_rectale	Diabetes	30
57aefa5d0a0056772a9e3e71c4ddec1b	f__Lactobacillaceae; g__Lactobacillus; s__Lactobacillus_fermentum	Diabetes	29
